# Inhibition of microbiota-dependent TMAO production attenuates chronic kidney disease in mice

**DOI:** 10.1038/s41598-020-80063-0

**Published:** 2021-01-12

**Authors:** Wenchao Zhang, Aika Miikeda, Jonathan Zuckerman, Xun Jia, Sarada Charugundla, Zhiqiang Zhou, Karolina Elżbieta Kaczor-Urbanowicz, Clara Magyar, Fangfei Guo, Zeneng Wang, Matteo Pellegrini, Stanley L. Hazen, Susanne B. Nicholas, Aldons J. Lusis, Diana M. Shih

**Affiliations:** 1 The Key Laboratory of Cardiovascular Remodeling and Function Research, Chinese Ministry of Education, Chinese National Health Commission and Chinese Academy of Medical Sciences, The State and Shandong Province Joint Key Laboratory of Translational Cardiovascular Medicine, Department of Cardiology, Qilu Hospital, Cheeloo College of Medicine, Shandong University, Jinan, 250012 Shandong China; 2 Department of Critical Care Medicine, Qilu Hospital, Cheeloo College of Medicine, Shandong University, Jinan, 250012 Shandong China; 3 Division of Cardiology, Department of Medicine, University of California, 10833 Le Conte Avenue, A2-237 CHS, Los Angeles, CA 90095-1679 USA; 4 Department of Pathology and Laboratory, University of California, Los Angeles, CA 90095 USA; 5 Department of Cardiovascular and Metabolic Sciences, Lerner Research Institute, Cleveland Clinic, Cleveland, OH 44195 USA; 6 Division of Oral Biology and Medicine, Center for the Health Sciences, UCLA School of Dentistry, Center for Oral and Head/Neck Oncology Research, UCLA Section of Oral Biology, University of California, 10833 Le Conte Ave, Box 951668, Los Angeles, CA 90095 USA; 7 UCLA Institute for Quantitative and Computational Biosciences, University of California, 611 Charles E. Young Drive Boyer Hall 570, Box 951570, Los Angeles, CA 90095 USA; 8 Translational Pathology Core Laboratory, University of California, Los Angeles, CA 90095 USA; 9 Molecular, Cell, & Developmental Biology, University of California, Los Angeles, CA 90095 USA; 10 Department of Cardiovascular Medicine, Heart, Vascular and Thoracic Institute, Cleveland Clinic, Cleveland, OH 44195 USA; 11 Department of Medicine/Division of Nephrology, University of California, Los Angeles, CA 90095 USA

**Keywords:** Cell biology, Molecular biology, Cardiology, Diseases, Nephrology

## Abstract

Patients with chronic kidney disease (CKD) have elevated circulating levels of trimethylamine N-oxide (TMAO), a metabolite derived from gut microbes and associated with cardiovascular diseases. High circulating levels of TMAO and its dietary precursor, choline, predict increased risk for development of CKD in apparently healthy subjects, and studies in mice fed TMAO or choline suggest that TMAO can contribute to kidney impairment and renal fibrosis. Here we examined the interactions between TMAO, kidney disease, and cardiovascular disease in mouse models. We observed that while female hyperlipidemic apoE KO mice fed a 0.2% adenine diet for 14 weeks developed CKD with elevated plasma levels of TMAO, provision of a non-lethal inhibitor of gut microbial trimethylamine (TMA) production, iodomethylcholine (IMC), significantly reduced multiple markers of renal injury (plasma creatinine, cystatin C, FGF23, and TMAO), reduced histopathologic evidence of fibrosis, and markedly attenuated development of microalbuminuria. In addition, while the adenine-induced CKD model significantly increased heart weight, a surrogate marker for myocardial hypertrophy, this was largely prevented by IMC supplementation. Surprisingly, adenine feeding did not increase atherosclerosis and significantly decreased the expression of inflammatory genes in the aorta compared to the control groups, effects unrelated to TMAO levels. Our data demonstrate that inhibition of TMAO production attenuated CKD development and cardiac hypertrophy in mice, suggesting that TMAO reduction may be a novel strategy in treating CKD and its cardiovascular disease complications.

## Introduction

Previous studies by our groups identified a novel dietary/meta-organismal pathway for cardiovascular disease (CVD) whereby bacterial metabolism of dietary choline and L-carnitine in the intestine leads to an intermediate metabolite, trimethylamine (TMA), which is absorbed from the gut and subsequently oxidized by hepatic flavin-containing monooxygenases (FMO) to generate trimethylamine N-oxide (TMAO)^1–3^. Romano et al. identified eight bacterial species from human intestine representing two different phyla (*Firmicutes* and *Proteobacteria),* which can convert choline to TMA in vitro^4^. A more recent study quantified and characterized bacterial genes encoding enzymes responsible for TMA production, including choline-TMA lyase (*CutC*), carnitine oxygenase (*CntA*) and betaine reductase (*GrdH*) in 89 fecal samples derived from various mammals^5^. The study showed that bacteria harboring *CutC* and *GrdH* are predominately affiliated with various taxa within *Firmicutes,* whereas *CntA* comprised sequences primarily linked to *Escherichia*^5^. In humans, plasma TMAO levels are elevated in patients with CVD compared to healthy controls, and exhibit a dose-dependent relationship with severity of atherosclerotic disease, and are associated with a ~ twofold increased risk for major adverse cardiac events (MACE), including death, myocardial infarction (MI), and stroke, independent of traditional CVD risk factors, renal function, and medications^6–8^. Multiple studies have replicated the association of TMAO with CVD and mortality^8,9^, including in patients with chronic heart failure^8,10^, diabetes mellitus^11^ and renal disease^12,13^. Multiple meta analyses confirm an overall positive association between circulating TMAO levels and CVD and mortality risks^8,14^, though not all studies have reported the same positive relationships^9^. Feeding mice with choline or TMAO leads to significant increases in plasma TMAO levels, thereby promoting atherosclerosis and thrombosis^2,15^. Non-lethal inhibition of gut microbial TMA production has recently been shown to decrease atherosclerosis^16^ and thrombosis potential^17^, and benefit tissue remodeling changes in preclinical models of heart failure^18^ and isoproterenol infusion-driven cardiorenal disease in mice^19^. Thus, there is growing potential for the use of inhibitors that target the gut microbiome as a novel therapeutic strategy for the treatment of cardiovascular and metabolic diseases.

Chronic kidney disease (CKD) is a serious public health problem that affects 15% (37 million) of United States (US) adults (Center for Disease Control and Prevention. Chronic Kidney Disease Surveillance System—US 2020. Available from: http://www.cdc.gov/ckd) and is associated with premature death, primarily due to CVD^20,21^. TMAO levels in patients with CKD are elevated^12,13,22–24^, likely due to impaired renal excretion of TMAO^12,23^ and possibly due to altered gut microbiome^22^. In addition, mice fed with choline or TMAO demonstrate significantly increased renal fibrosis and impaired renal function^12^, suggesting that TMAO is not merely a marker of reduced renal function, but a potential cause of CKD. Furthermore, choline and TMAO feeding in mice is associated with increased phosphorylation of SMAD family member 3 (Smad3) in the kidney^12^, which is an important regulator of the profibrotic transforming growth factor β (TGF-β)/Smad3 signaling pathway during fibrotic kidney disease^25^. In a previous study we showed that choline feeding increases TMAO levels and promotes inflammatory responses in arterial vascular cells through the activation of the nuclear factor-kappa B (NF-κB) signaling pathway^26^. In addition, TMAO has been suggested to promote CVD through several other pro-atherogenic mechanisms, including foam cell formation, reverse cholesterol transport, platelet responsiveness/thrombosis, activation of the NLR family pyrin domain containing 3 (NLRP3) inflammasome, and changes in tissue cholesterol, sterol, and bile acid metabolism^2,3,15,27–29^. These studies support the notion that the increased CVD risk associated with CKD may arise in part from elevated TMAO levels.

Here, we investigate how inhibition of gut microbiota-dependent TMA production affects the development of adenine-induced CKD and CVD traits in hyperlipidemic apolipoprotein E (apoE) knockout (KO) mice. Our results indicate that TMAO promotes CKD, and that in the context of CKD this contributes to cardiac hypertrophy.

## Results

### Inhibition of TMAO production attenuates chronic kidney disease induced by adenine feeding

**Figure 1:**
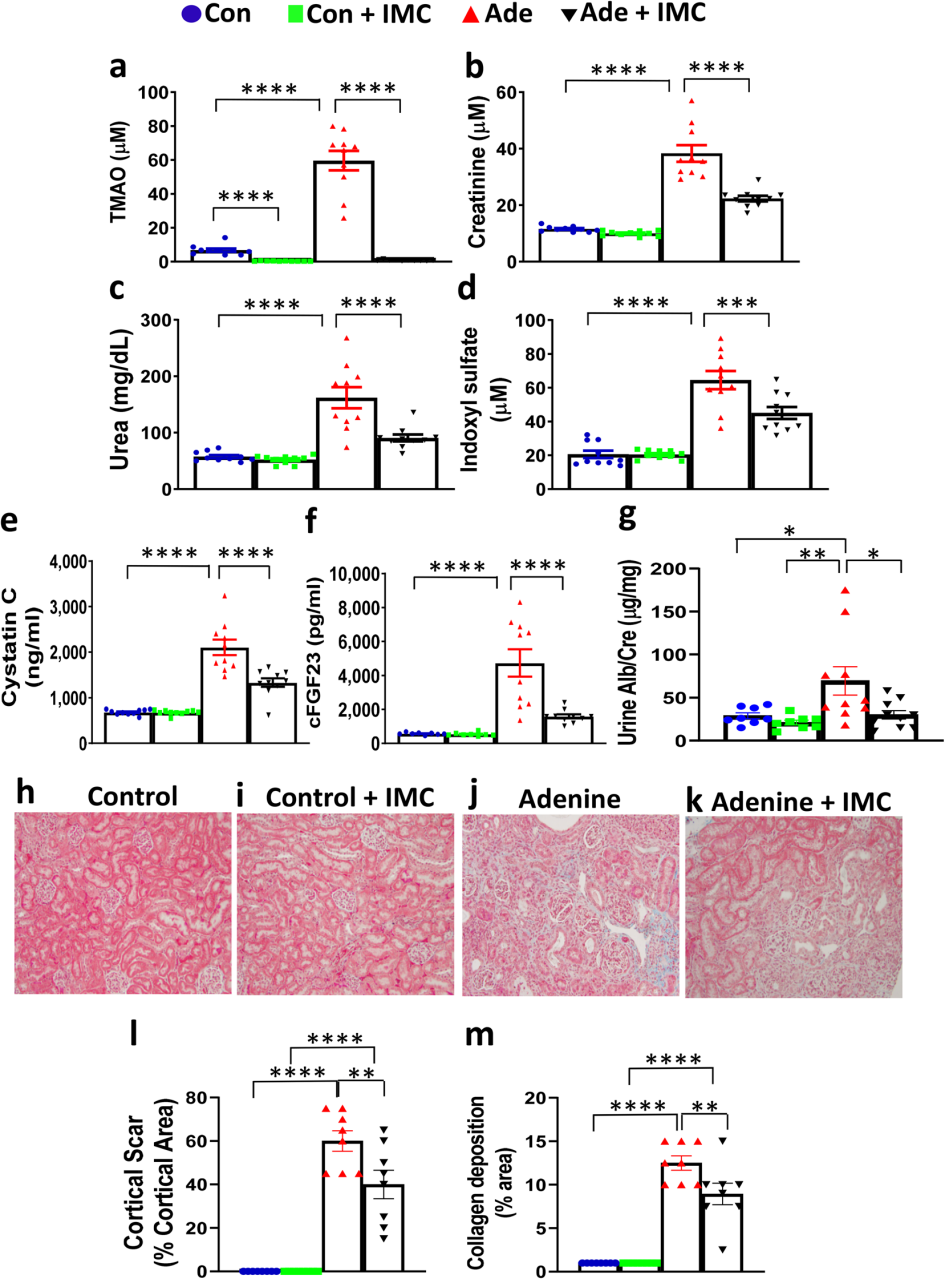
Inhibition of TMAO production attenuates chronic kidney development induced by adenine feeding. Female apoE KO mice (n = 10 per group) were fed control (Con), control + IMC (Con + IMC), adenine (Ade), or adenine + IMC (Ade + IMC) diets for 14 weeks before measurements of traits. Plasma levels of (**a**) TMAO, (**b**) creatinine, (**c**) Urea, (**d**) Indoxyl sulfate, (**e**) Cystatin C, and (**f**) c-terminal FGF23 (cFGF23) are shown. (**g**) Urine albumin (Alb) to creatinine (Cre) ratios are shown. Representative kidney sections stained with Masson’s trichrome are shown in (**h**–**k**). Quantification of kidney pathology, (**l**) cortical scar, and (**m**) collagen deposition, in the kidney sections of all 4 groups are shown. Individual values, means, and standard errors are shown. Sample sizes were n = 10/group for (**a**–**f**), n = 8–10/group for (**g**), and n = 8 kidneys/group for (**l**,**m**). For (**a**–**f**,**l**,**m**), post hoc multiple comparisons analysis was performed after significant two-way ANOVA. Symbols: *p < 0.05, **p < 0.01, ***p < 0.001, and ****p < 0.0001.

ApoE KO mice, instead of wild-type mice, were chosen for this study since we aimed to study atherosclerosis and these mice develop hypercholesterolemia and atherosclerosis spontaneously without dietary intervention^30^. Furthermore, CKD induced by 5/6 nephrectomy (5/6Nx) has been shown to increase atherosclerosis in apoE KO mice^31–33^. Female mice were chosen for the study instead of males since our preliminary data showed that male mice fed the adenine diet for 8 weeks lost a very substantial amount of body weight (19%) and appeared unhealthy for further characterization. It appears that sex hormones may be the cause of greater susceptibility of male kidneys to progressive renal injury in mice^34,35^. Therefore, one-month old female apoE KO mice were fed standard chow (control; Con) or adenine (Ade) diets with or without iodomethylcholine (IMC; see Methods) for 14 weeks to examine the effects of reduced TMAO on CKD and atherosclerosis. At the end of the 14-week feeding period, the body weights of mice receiving either Ade or Ade + IMC were significantly decreased compared to either Con or Con + IMC groups, whereas there were no significant differences in body weight between Con and Con + IMC or between Ade and Ade + IMC groups (Supplemental Fig. S1). The decreased body weights observed in the Ade and Ade + IMC groups compared to the control groups were not due to decreased food consumption since there were no significant differences in food consumption among the 4 groups of mice (Supplemental Fig. S1). Similar food consumption between the Ade and Ade + IMC mice suggested that similar amounts of adenine were ingested by these two groups of mice. Plasma TMA levels of the Con + IMC group showed a trend of a decrease compared to the Con group (0.08 μM vs. 0.15 μM, p = 0.15, Supplemental Fig. S1). Also, there was a trend of a decrease in TMA levels in the Ade + IMC group compared to the Ade group (0.08 μM vs. 0.14 μM, p = 0.15). Plasma TMAO levels of the Ade group were 8.8-fold higher than the Con group (60 μM vs. 6.8 μM, p < 0.0001, Fig. 1a). IMC dramatically decreased TMAO levels (0.8 μM) in Ade + IMC group compared to the Ade group (p < 0.0001, Fig. 1a). In addition, TMAO levels of Con + IMC group were significantly decreased compared to the Con group (0.4 μM vs. 6.8 μM, p < 0.0001, Fig. 1a). These data demonstrated the effectiveness of IMC in blocking TMAO production in mice fed either a control or an adenine diet.

We next monitored multiple circulating markers of kidney function, including creatinine, urea, cystatin C, and fibroblast growth factor 23 (FGF23). Notably, all markers were significantly elevated in the Ade group compared to the Con group, confirming renal injury and reduced function in the adenine fed mice. Importantly, mice in the Ade + IMC group showed significantly reduced circulating levels of all renal injury markers compared to the Ade group: 41% decrease in creatinine (p < 0.0001), 41% decrease in urea (p < 0.0001), 37% decrease in cystatin C (p < 0.0001), and 64% decrease in FGF23 (p < 0.0001) (Fig. 1b,c,e,f), demonstrating that IMC attenuated CKD induced by adenine feeding. In additional studies, circulating levels of a uremic toxin, indoxyl sulfate, were found to be increased threefold in the Ade group compared to the Con group (Fig. 1d). However, when the gut microbiota targeting inhibitor IMC was provided, there was a significant 31% reduction in indoxyl sulfate levels in the Ade + IMC group versus Ade group (p < 0.001, Fig. 1d). The adenine diet-induced CKD mouse model simulates multiple features of CKD including development of microalbuminuria. We therefore examined urine albumin-to-creatinine ratio (UACR) in the mouse groups and noted a significant increase in the Ade group (compared to the Con and Con + IMC groups; Fig. 1g), indicating impaired kidney function in the former. The Ade + IMC group exhibited significantly decreased UACR (57% decrease, p = 0.03) compared to the Ade group, demonstrating significant improvement in kidney function in the former (Fig. 1g). In fact, there were no significant differences in UACR among the Con, Con + IMC, and Ade + IMC groups (Fig. 1g).

To examine tissue remodeling during CKD, histological sections of kidney were stained with Masson’s trichrome and quantitated for cortical scar area and collagen deposition. Kidney sections of both Con and Con + IMC groups appeared normal (Fig. 1h,i), whereas those of the Ade and Ade + IMC groups showed cortical scar and collagen deposition (Fig. 1j,k). Quantification of the pathological changes revealed no cortical scar and less than 1% collagen deposition in the Con and Con + IMC groups (Fig. 1l,m). There were significant decreases in cortical scar (by 33%, p = 0.003) and collagen deposition (by 28%, p = 0.004) in the Ade + IMC group compared to the Ade group (Fig. 1l,m). There was no global glomerulosclerosis, vascular, or perivascular fibrosis in any group. These data demonstrated that IMC not only significantly decreased circulating levels of kidney disease markers but also decreased renal pathological changes associated with adenine feeding and improved kidney function.

### IMC supplementation results in a kidney gene expression profile consistent with decreased inflammation and fibrosis following adenine feeding

**Figure 2:**
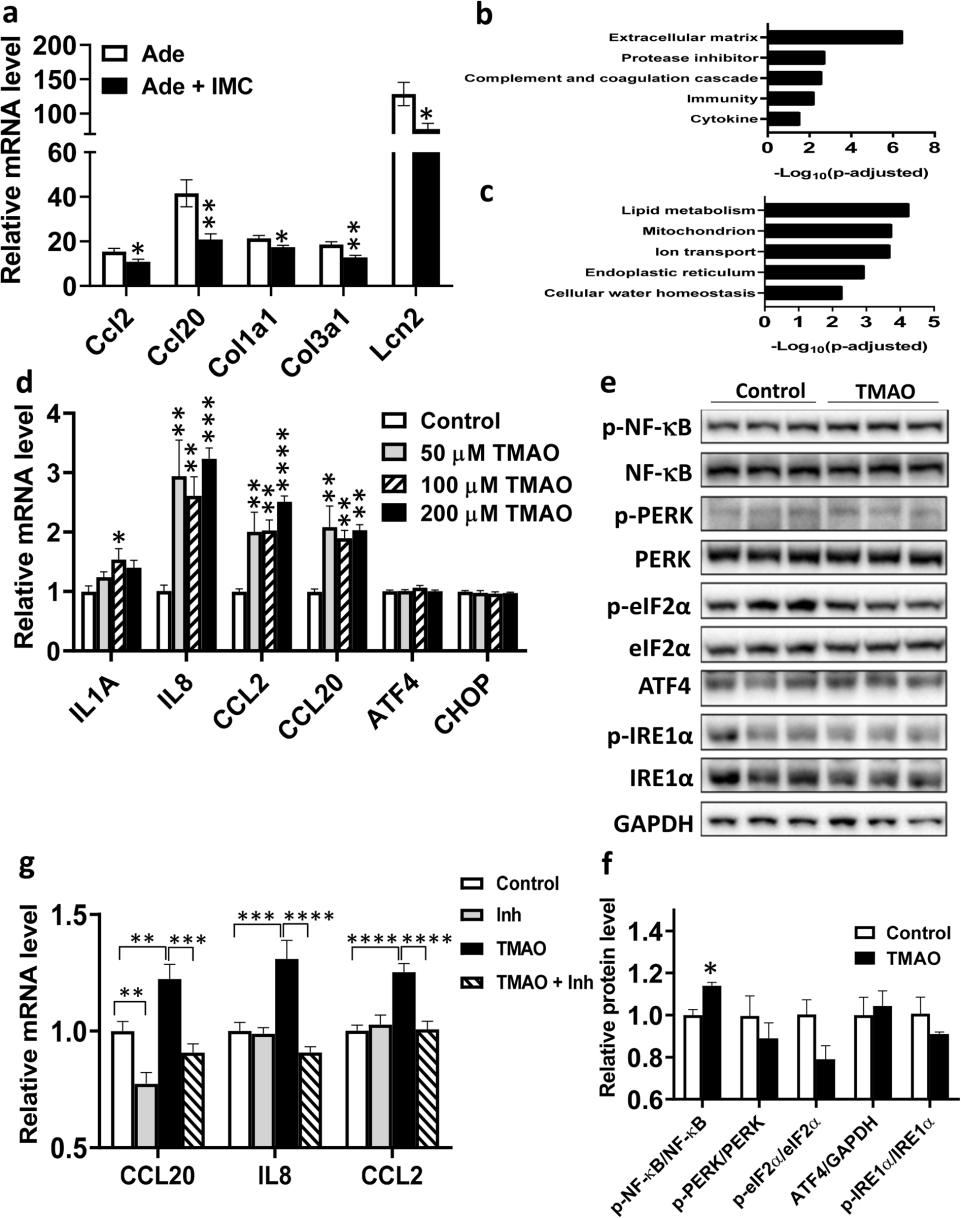
Inhibition of TMAO generation decreases inflammation and fibrosis in the kidneys of adenine diet-induced CKD. Gene expression analysis of kidney samples from the same mice described in Fig. 1 were performed using qPCR (**a**), n = 8 to 9 per group, and RNA-seq (**b**,**c**). Based on the RNA-seq data, pathway enrichment analysis was performed to identify down-regulated (**b**), and up-regulated (**c**) functional clusters in the kidneys of Ade + IMC group compared to the Ade group (n = 6 per group). Gene expression (**d**, n = 4–5/group) and immunoblotting (**e**,**f**, n = 3/group) analysis of MDCK II kidney epithelial cells treated with control, and various doses of TMAO (**d**) or 200 μM of TMAO (**e**,**f**) for 48 h. (**g**) MDCKII cells were treated with control media, 100 nM NF-κB inhibitor IV (Inh), 200 μM TMAO (TMAO), or 200 μM TMAO + 100 nM NF-κB inhibitor IV (TMAO + Inh) for 48 h before gene expression analysis (n = 9 for each group). For (**a**,**f**), two tailed Student’s t-test was performed. Symbols: *p < 0.05, and **p < 0.01. For (**d**) Post hoc Tukey’s multiple comparisons test was performed after significant one-way ANOVA. Symbols: *p < 0.05, **p < 0.01, ***p < 0.001, and ****p < 0.0001 compared to the control group. For (**g**) Post hoc analysis was performed after significant two-way ANOVA. Symbols: **p < 0.01, ***p < 0.001, and ****p < 0.0001.

qPCR analysis revealed that adenine treatment significantly increased the mRNA levels of inflammatory and fibrosis genes in the kidney by 15- to 100-fold compared to the Con group (Fig. 2a). IMC supplementation significantly decreased renal expression of inflammatory genes, C–C motif chemokine ligand 2 (Ccl2, p < 0.05), C–C motif chemokine ligand 20 (*Ccl20,* p < 0.01), and lipocalin-2 (*Lcn2,* p < 0.05), and fibrosis genes, collagen type I alpha 1 chain (*Col1a1,* p < 0.05), and collagen type III alpha 1 chain (*Col3a1,* p < 0.01), caused by adenine feeding (Fig. 2a). RNA-seq analysis revealed 627 down-regulated and 584 up-regulated genes in the kidneys of Ade + IMC group compared to the Ade group (data not shown). Pathway analysis of the downregulated genes showed the enrichment of extracellular matrix, protease inhibitor, complement and coagulation cascade, immunity, and cytokine clusters (Fig. 2b and Supplemental Table S1). The protease inhibitor functional cluster includes: WAP 4-disulfide core domain-2 (*Wfdc2*), plasminogen activatorinhibitor-1 (PAI-1, encoded by *Serpine1*), tissue inhibitor of metalloproteinase 1 (*Timp1*) and 10 other genes (Supplemental Table S1). Overall, our data suggested that IMC supplementation attenuated the pathological processes, such as fibrosis and inflammation, induced by adenine feeding. The up-regulated functional clusters include: lipid metabolism, mitochondria, ion transport, endoplasmic reticulum, and cellular water homeostasis (Fig. 2c and Supplemental Table S2), indicating an improved metabolic state associated with IMC supplementation.

### TMAO treatment induced inflammation via NF-κB activation in kidney epithelial cells

To better understand the mechanism of the action of TMAO in the kidney, we quantified the expression of inflammatory and unfolded protein response (UPR) genes in MDCK II kidney epithelial cells in response to various doses of TMAO after 48 h. We observed that TMAO (as low as 50 μM up to 200 μM) significantly increased the mRNA levels of the inflammatory genes: interleukin 8 (*IL8*), *CCL2*, and *CCL20* by two to threefold compared to the control group, whereas the mRNA levels of genes involved in UPR, activating transcription factor 4 (ATF4) and CHOP (DNA damage inducible transcript 3, *DDIT3*) were similar between the TMAO treated and control cells (Fig. 2d). Immunoblotting analysis revealed that the NF-κB pathway is activated by TMAO treatment, as evidenced by significant increases in both the phosphorylation of the NF-κB p65 subunit (Fig. 2e) and the p-NF-κB p65 to total NF-κB p65 ratio (Fig. 2f). In contrast, under the conditions examined, TMAO treatment did not activate proteins in the UPR pathways, including protein kinase R-like endoplasmic reticulum kinase (PERK), eukaryotic initiation factor 2 alpha (eIF2α), ATF4, and inositol-requiring enzyme 1 alpha (IRE1α), in MDCK II cells (all p > 0.09; Fig. 2e,f). The presence of an NF-κB inhibitor, NF-κB inhibitor IV, completely abolished the stimulatory effect of TMAO on the expression of *CCL20* (p < 0.001), *IL8* (p < 0.0001), and *CCL2* (p < 0.0001) (Fig. 2g), suggesting the importance of NF-κB in mediating the inflammatory effect of TMAO. TMAO treatment also significantly induced the expression of the inflammatory molecules, CCL2 and tumor necrosis factor-alpha (TNF-α), in normal human primary renal proximal tubule epithelial cells (RPTEC) (Supplemental Fig. S2).

### Effects of IMC on cardiovascular disease related traits in mice fed the adenine diet

**Figure 3:**
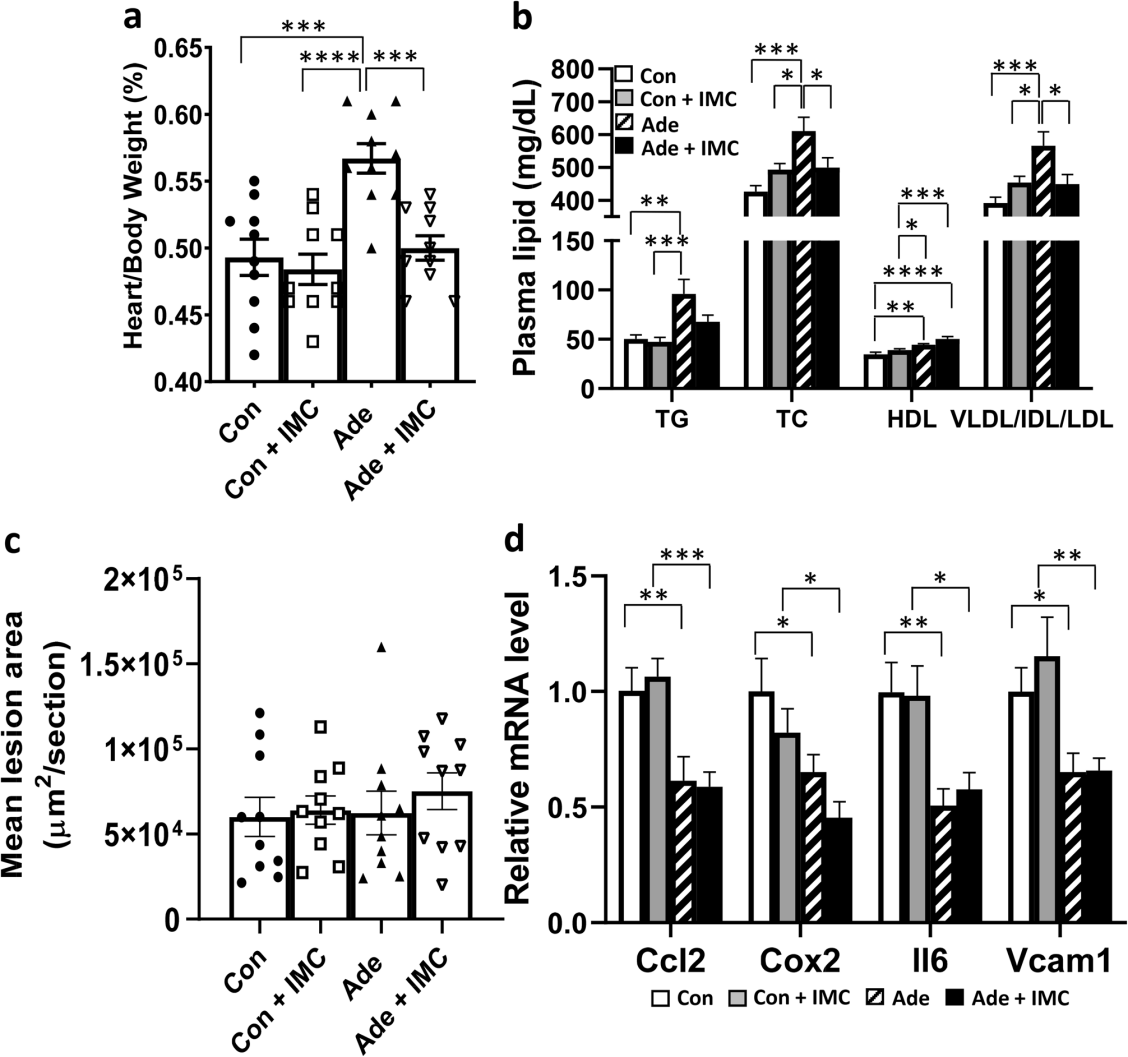
IMC supplementation protects against myocardial hypertrophy and dyslipidemia associated with adenine diet-induced CKD. Data obtained from tissue samples of the same animals described in Fig. 1 are presented in this figure, n = 10 per group. (**a**) Heart weight/body weight ratios, (**b**) Plasma levels of triglyceride (TG), total cholesterol (TC), HDL cholesterol (HDL), and VLDL/IDL/LDL cholesterol (VLDL/IDL/LDL), (**c**) mean atherosclerotic lesion area at the aortic root, and (**d**) aortic gene expression analysis by qPCR are shown. For data presented in all panels, post hoc multiple comparisons analysis was performed after significant two-way ANOVA. Symbols: *p < 0.05, **p < 0.01, ***p < 0.001, and ****p < 0.0001.

Adenine-fed mice exhibited significantly increased heart weight/body weight ratio (a surrogate marker for myocardial hypertrophy^36^) compared to Con, Con + IMC, and Ade + IMC groups (Fig. 3a), whereas no differences in heart weight/body weight ratio were observed among the Con, Con + IMC, and Ade + IMC groups (Fig. 3a). These data suggested that adenine feeding induced heart hypertrophy in mice—likely as a result of CKD—and cardiac hypertrophy was blocked by IMC treatment.

Adenine diet also worsened the hyperlipidemia phenotypes in apoE KO mice with increased plasma triglyceride, total cholesterol, and VLDL/IDL/LDL cholesterol levels compared to Con and Con + IMC groups (Fig. 3b). In contrast, the Ade + IMC group exhibited significant decreases in plasma total cholesterol (by 18%, p < 0.05) and VLDL/IDL/LDL cholesterol (by 21%, p < 0.05) levels compared to the Ade group, suggesting improved plasma lipoprotein profile (Fig. 3b). Surprisingly, both the Ade and Ade + IMC groups had significantly increased HDL cholesterol levels compared to the Con and Con + IMC group (Fig. 3b). Unexpectedly, there were no significant differences in mean atherosclerotic lesion area at the aortic root region among the 4 groups of mice (Fig. 3c). Gene expression analysis of the aorta showed significantly reduced mRNA levels of *Ccl2*, Cox2 (prostaglandin-endoperoxide synthase 2, *Ptgs2*), interleukin 6 (*Il6*), and vascular cell adhesion molecule 1 (*Vcam1*) in the Ade and Ade + IMC groups compared to those of the Con and Con + IMC groups (Fig. 3d), suggesting decreased inflammation in the aortas of mice of the former groups, despite induction of CKD.

## Discussion

In this study, we demonstrated that targeted inhibition of bacterial choline TMA lyase activity dramatically decreased circulating TMAO levels and multiple indices of renal function and adverse remodeling in an adenine-induced CKD mouse model. In the adenine-fed apoE KO model where renal function declined, multiple markers of injury were increased and adverse histopathological changes occurred. Provision of IMC blocked these adverse effects, normalizing renal function and histology, and improving the many CKD markers including creatinine, urea, cystatin C, FGF23, TMAO, and UACR. Of importance, we observed suppression of TMAO with the non-lethal inhibitor significantly decreased kidney cortical scar and fibrosis area, and also decreased expression of multiple inflammatory and fibrosis genes. RNA-seq analysis in the kidneys of Ade + IMC group compared to the Ade group revealed downregulation of pathways involved in extracellular matrix, immunity, and cytokine, and upregulation of pathways involved in lipid metabolism, mitochondria, endoplasmic reticulum, ion transport and water homeostasis. Therefore, gut microbiota dependent inhibition of TMA generation using IMC attenuated CKD development in the host and preserved kidney function. Recent studies demonstrated halomethylcholine based microbial choline TMA lyase inhibitors block TMAO generation in the host and elicit protective effects on atherosclerosis, thrombosis, and heart failure^16–18^. In agreement with our findings, in male wild-type C57BL/6 mice, IMC has been shown to inhibit TMA and TMAO production and prevent choline diet-induced renal functional decline and adverse remodeling in a chronic sympathetic-driven (isoproterenol infusion) model of CKD^19^. Our study provides further evidence that inhibition of a gut microbe pathway (the TMAO metaorganismal pathway) can have a profound effect on the host—this time demonstrating marked inhibition in progression of CKD in an adenine feeding model.

Chronic kidney disease is associated with altered gut microbiome in humans and animals^22,37,38^. Also, CKD patients tend to have higher percentages of opportunistic pathogens and decreased percentages of beneficial microbes in the gut^22^. Furthermore, CKD patients had increased plasma TMAO levels and significantly increased abundance of bacterial genes related to TMA production in the intestine compared to healthy controls^22^. A fecal microbiota transplantation study showed that antibiotic-treated mice receiving fecal samples from CKD patients had significantly higher plasma TMAO levels and different composition of gut microbiota than did mice receiving fecal samples from healthy subjects^22^. These data suggest that elevated plasma TMAO level associated with CKD is caused at least in part by increased abundance of TMA producing bacteria in the gut. Therefore, our strategy of non-lethal inhibition of gut microbial TMA production is likely to be beneficial to CKD patients in slowing the progression of CKD and decreasing CVD risk.

Our cell culture studies demonstrated that TMAO treatment induced the expression of inflammatory genes by activation of NF-κB in kidney epithelial cells, similar to our previous observation in human aortic endothelial and smooth muscle cells^26^. Therefore, we postulate that TMAO exacerbates the development of CKD, at least in part via activation of NF-κB pathway in relevant cell types in the kidney. Unlike the data previously reported in primary hepatocytes^39^, we failed to observe activation of PERK or other UPR pathways in TMAO-treated kidney epithelial cells. This may have resulted from differences in cell types, experimental conditions, or other factors. Whether PERK plays a role in recognition of TMAO as a receptor involved in phenotypes monitored is unclear, and merits further investigation. Thus far, only glucose related phenotypes linked to TMAO have been implicated in being transmitted in part via the involvement of PERK and other UPR genes^39^.

Increased NF-κB activation has been observed in the kidneys of patients with type 2 diabetic nephropathy^40^. Inhibition of NF-κB signaling through feeding of a chemical inhibitor, pyrrolidine dithiocarbamate, has been shown to decrease inflammation and CKD development in various animal models, including: type 1 diabetes^41^, adenine overload^42^, and 5/6 nephrectomy^43^. Our findings that TMAO activated NF-κB, leading to increased expression of inflammatory genes in kidney epithelial cells, suggest NF-κB activation as one of the mechanisms by which TMAO may promote CKD. We postulate that activation of NF-κB by TMAO leads to increased inflammation which is a major driver of fibrosis^44,45^. In fact we observed significantly decreased expression of genes belonged to the extracellular matrix pathway in the kidneys of Ade + IMC group compared to those of Ade group (Fig. 2b and Supplemental Table S1), supporting our hypothesis.

We observed significantly decreased expression of Wfdc2, PAI-1/Serpine1, Timp1 and 10 other genes of the protease inhibitor functional cluster in the kidneys of Ade + IMC group compared to those of the Ade group (Supplemental Table S1). Wfdc2 is a serine protease inhibitor that is upregulated in human and mouse fibrotic kidneys and suppresses the degradation of type I collagen. Administration of Wfdc2 neutralizing antibodies accelerated collagen I degradation and inhibited fibrosis in various mouse models of renal disease, demonstrating a profibrotic role for Wfdc2^46^. PAI-1/Serpine1, a serine protease inhibitor, is thought to be profibrotic by impairing the turnover and degradation of extracellular matrix proteins. Furthermore, PAI-1 plays an important role in the interstitial recruitment of inflammatory macrophages and myofibroblasts in response to chronic kidney injury^47,48^. Timp1 inhibits the activities of matrix metalloproteinases. Timp1 deficiency in mice is associated with significantly reduced myocardial fibrosis in models of cardiomyopathy in vivo^49^. Timp1 also mediates an association between CD63 (cell surface receptor for TIMP1) and integrin β1 on fibroblasts, initiates activation and nuclear translocation of Smad2/3 and β-catenin, leading to de novo collagen synthesis^49^. Therefore, the decreased expression of these protease inhibitor genes in the kidneys of the Ade + IMC mice compared to those of the Ade mice may explain, in part, the decreased collagen deposition in kidneys of the former (Fig. 1m).

We observed significantly decreased heart weight/body weight ratios in the Ade + IMC group as compared to the Ade group, suggesting decreased cardiac hypertrophy in the former. FGF23 is a bone derived hormone with a central role in the regulation of phosphate homeostasis^50^. CKD patients exhibit elevated circulating levels of FGF23, which has been shown to induce left ventricular hypertrophy^51^ that can lead to heart failure. We observed significantly increased circulating FGF23 levels in the adenine-fed mice compared to the control mice, whereas IMC supplementation significantly decreased FGF23 levels induced by adenine feeding. Therefore, the decrease in heart weight/body weight ratios in the Ade + IMC group compared to the Ade group could be attributed in part to decreased FGF23 levels.

CKD induced by 5/6 nephrectomy (5/6Nx) has been shown to increase atherosclerosis in apoE KO mice^31–33^. Adenine feeding, a non-surgical approach to induce CKD, induces tubulointerstitial nephropathy in mice^52^. It also induces cardiac hypertrophy and decreases cardiac function in mice^53^. Surprisingly, atherosclerotic lesion areas at the aortic root of the Ade group were not increased compared to the Con or Con + IMC groups. In fact, gene expression analysis of the aortas revealed that expression of inflammatory genes was decreased in the Ade and Ade + IMC groups compared to the Con and Con + IMC groups, suggesting that, by unknown mechanisms, adenine feeding decreased inflammation locally in the aorta despite induction of CKD. Adenine is the precursor of adenosine that is known to play a role in the control of inflammation^54^. One of the adenosine receptors, A2B adenosine receptor, is expressed in the vasculature and macrophages and protects against inflammation and excessive vascular adhesion^55^. We hypothesize that adenine feeding may lead to increased adenosine level in the vasculature, leading to increased activation of A2B adenosine receptor, decreased vascular inflammation and atherosclerosis. Therefore, our data suggest that although adenine feeding is suitable for studying CKD development and CKD-induced cardiac hypertrophy and dysfunction, the model did not permit investigation of the relationship between CKD and atherosclerosis.

In summary, our study demonstrated that inhibition of TMAO production in an adenine-induced model of CKD significantly attenuated the development of CKD and cardiac hypertrophy. Our cell culture studies support the concept that TMAO promotes development of CKD by activation of NF-κB, leading to aggravated inflammation. Inhibition of TMAO production provides a novel strategy in the treatment CKD and reno-cardiac syndrome.

## Materials and methods

### Animal studies

All animal experiments were approved by the UCLA Animal Care and Use Committees, in accordance with PHS guidelines. One-month-old female apoE KO mice on C57BL/6J background (stock number: 002052, Jackson laboratory, Bar Harbor, ME), n = 10/group, were fed ab libitum the following diets from Envigo (Madison, WI) for 14 weeks before tissue collection: (1) chow diet (Con, TD.110846), (2) chow diet + 0.06% iodomethylcholine (IMC) (Con + IMC, TD.170932), (3) chow diet + 0.2% adenine (Ade, TD.170076), and (4) chow diet + 0.2% adenine + 0.06% IMC (Ade + IMC, TD.170093). At the 12th week of diet feeding, mice were individually placed in metabolic cages for collection of urine for 24 h. At the end of the diet feeding period, mice were fasted for 4 h before blood and tissue collection.

### Biochemical assays

Plasma TMA, TMAO and creatinine levels were determined by mass spectrometry as previously described^2^. Indoxyl sulfate was determined by Shimadzu 8050 LC/mass spectrometer by using a phenyl column (250 × 2 mm, Thermo Scientific) and resolving by a gradient generated between A, 0.2% formic acid in water and B, 0.2% formic acid in methanol with the initial two minutes at 0% B, then linearly rose to 90% B over 7.3 min, then to 100% B over 0.5 min and held for 3 min, then back to 0% B and equilibrium for 3 min. Indoxyl sulfate (Cayman Chemical, Ann Arbor, MI) and its respective internal standard, indoxyl sulfate (ring-d4) were monitored using ESI in positive-ion mode with multiple reaction monitoring (MRM) of precursor and characteristic product ion transitions as: m/z 212 → 80 for indoxyl sulfate, 216 → 80 for indoxyl sulfate (ring-d4). Sample preparation for the measurement of indoxyl sulfate followed the same procedure as the measurement of TMAO with 1 μM indoxyl sulfate (ring-d4) added to the TMAO internal standard mix in methanol^2^. Plasma total cholesterol, HDL cholesterol, and triglyceride levels were determined as previously described^27^. Plasma urea levels were determined using a colorimetric assay (Bioassay Systems, Hayward, CA). Plasma FGF23 (Mouse/Rat FGF-23 (C-Term) ELISA kit, Quidel, San Diego, CA) and cystatin C (R&D Systems, Minneapolis, MN) levels were determined by ELISA. Urine albumin and creatinine levels were determined using an ELISA kit (Exocell, Philadelphia, PA) and a colorimetric assay kit (Exocell), respectively for calculation of UACR.

### RNA isolation and quantitative RT-PCR analyses

Total RNA was isolated from tissue samples using the miRNA isolation kit (Qiagen, Germantown, MD) according to the protocol provided by the manufacturer. The cDNA was synthesized using the High Capacity cDNA Reverse Transcription Kit (Applied Biosystems, Foster City, CA). Quantitative PCR was performed using gene-specific primers (Supplemental Table S3) and the Roche SYBR green master mix. Samples were run on a LightCycler 480 II system (Roche, Pleasanton, CA) and analyzed using the Roche LightCycler 1.5.0 software^26^. All qPCR targets were quantified based on standard curves ran on the sample plate. The mRNA levels of specific genes were normalized to the mRNA levels of housekeeping genes of the same sample. The mRNA levels of housekeeping genes eukaryotic translation initiation factor 2A (*Eif2a*, Fig. 2a), glyceraldehyde 3-phosphate dehydrogenase (*GAPDH*, Fig. 2d,g), ribosomal protein L13a (*Rpl13a*, Fig. 3d), and β2 microglobulin (*B2M*, Supplemental Fig. S2) were used for normalization.

### Kidney histology

Histologic Sections (5 μm) from formalin fixed paraffin embedded mouse kidneys (8 kidneys per group) were stained with Masson’s trichrome and quantitated for cortical scar area and collagen deposition in a blinded fashion. The cortical scarring and collagen deposition were measured over the entire cortical tubulointerstitial area via semi-quantitative visual assessment performed by a renal pathologist.

### Atherosclerosis

Atherosclerotic lesions in the proximal aorta were quantitated as described^56^. Briefly, the heart was flushed with PBS and embedded in OCT. Frozen Sections (10 μM) were stained with Oil Red O and lesion area quantified every 6th section beginning at the aortic valves. The average of the first 10 scored sections was then used as a measure of lesion size.

### RNA-seq

Total RNA from kidney was isolated as described above. RNA libraries were prepared using the Illumina TruSeq kits (Illumina, San Diego, CA). Following barcoding, 24 samples per lane were sequenced on a HiSeq4000 using 50 bp single-end protocol. Reads were QC’d using FastQC in batch mode and mapped to the mouse genome (mm10) using STAR aligner version 2.3.1. The count data were normalized using DESeq2′s median of ratios method^57^. Differential expression analysis was performed using DEseq2^58^ with statistically significant genes called using adjusted p-value cutoffs of less than 0.1. Gene ontology analysis was performed using DAVID 6.8 (https://david.ncifcrf.gov/). The Benjamini–Hochberg method was used to obtain false discovery rates for enriched pathways.

### Cell culture studies

The Madin-Darby canine kidney II (MDCK II, from Sigma-Aldrich, St. Louis, MO) epithelial cells were cultured in DMEM (Corning, CA) supplemented with 5% fetal calf serum (Hyclone, Logan, UT) and 100 U/ml penicillin/100 μg/ml streptomycin (Gibco, Carlsbad, CA). For TMAO treatment, cells were plated in 6-well plates for 24 h, followed by treatment with TMAO (0, 50, 100, or 200 μM, Sigma-Aldrich) in the presence or absence of an NF-κB inhibitor (NF-κB inhibitor IV, 100 nM, Millipore, Burlington, MA) for 48 h before gene expression and immunoblotting analyses.

Normal human primary renal proximal tubule epithelial cells (RPTEC, ATCC, Manassas, VA) were grown in renal epithelial cell basal media (ATCC PCS400030) supplemented with renal epithelial cell growth kit (ATCC PCS400040), and 100 U/ml penicillin/100 μg/ml streptomycin (Gibco, Carlsbad, CA) according to the supplier’s protocol. For TMAO treatment, cells were plated in 6-well plates for 24 h, followed by treatment with TMAO (0, 50, 100, 200, or 400 μM, Sigma-Aldrich) for 48 h before gene expression analysis.

### Immunoblotting

Immunoblotting was performed using 20 μg of proteins from cell lysate per sample as previously described^26^. Primary antibodies against phospho-NF-κB p65, NF-κB p65, phospho-PERK, PERK, phospho-eIF2α, eIF2α, phospho-IRE1α, IRE1α, ATF4, and GAPDH (Cell Signaling Technology, Danvers, MA) were used in the experiments. After incubation with secondary antibody and extensive washing, blots were placed in Amersham ECL Prime Western Blotting Detection Reagent (GE Life Sciences). Blots were then imaged using the ChemiDoc MP system (Bio-Rad), and bands were quantified using ImageJ software (National Institutes of Health)^26^. Band intensities of phospho-NF-κB p65, phospho-PERK, phospho-eIF2α, and phospho-IRE1α were normalized by band intensities of total NF-κB p65, PERK, eIF2α, and IRE1α, respectively, of the same samples on the same blot after blot stripping and re-probing. Band intensities of ATF4 were normalized by band intensities of GAPDH of the same samples on the same blot.

### Statistical analyses

Two-tailed Student’s t-test was utilized to compare means between 2 groups. One-way ANOVA with Tukey’s multiple comparisons test was performed to analyze data presented in Fig. 2d and Supplemental Fig. S2. For experiments with a two-factorial design (data presented in Figs. 1a–g,l,m, 2g, 3a–d, and Supplemental Fig. S1a and S1b), a two-way ANOVA was performed to establish that not all groups were equal. The Holm–Sidak post hoc analysis was then used for specific between-group comparisons after statistical significance was established by ANOVA. Statistical analyses were performed in GraphPad Prism version 8.

## Supplementary Information

Supplementary Information

## Data Availability

All data are contained within the manuscript. The RNA-seq data presented in this publication have been deposited in NCBI's Gene Expression Omnibus^59^ and are accessible through GEO Series accession number GSE163276 (https://www.ncbi.nlm.nih.gov/geo/query/acc.cgi?acc=GSE163276).
