# Supplementary material for: Inhibition of microbiota-dependent TMAO production attenuates chronic kidney disease in mice
Source: Sci Rep. 2021 Jan 12;11:518. doi: 10.1038/s41598-020-80063-0 (PMC7804188; doi:10.1038/s41598-020-80063-0)

Supplemental information

Title: Inhibition of microbiota-dependent TMAO production attenuates chronic kidney disease in mice

Authors

Wenchao Zhang, Aika Miikeda, Jonathan Zuckerman, Xun Jia, Sarada Charugundla, Zhiqiang Zhou, Karolina Elżbieta Kaczor-Urbanowicz, Clara Magyar, Fangfei Guo, Zeneng Wang, Matteo Pellegrini, Stanley L. Hazen, Susanne B. Nicholas, Aldons J. Lusis, and Diana M. Shih

Supplemental Table S1. DAVID pathway analysis-enriched clusters of down-regulated genes in the kidney samples of Ade + IMC group compared to Ade group

Enrichment Cluster	Gene	log2 Fold Change	P value
**Extracellular matrix (30 genes)**	Adamts1	-0.52	1.21E-02
	Adamts16	-0.73	9.48E-03
	Adamts4	-0.50	1.14E-02
	Ctsd	-0.29	3.59E-02
	Clu	-0.58	1.84E-02
	Col3a1	-0.40	4.45E-03
	Col4a1	-0.32	1.78E-02
	Col5a2	-0.28	1.81E-02
	Col8a1	-0.48	1.01E-02
	Col12a1	-0.36	4.13E-02
	Col15a1	-0.38	3.83E-03
	Fbn2	-0.58	1.62E-02
	Lama1	-0.31	2.68E-02
	Ltbp2	-0.63	8.38E-03
	Lgals3	-0.40	3.45E-02
	Mmp12	-1.21	8.59E-05
	Mmp14	-0.30	2.22E-02
	Mmp20	-2.89	1.62E-02
	Mmp24	-1.31	3.61E-02
	Mmp7	-0.98	1.01E-02
	Rps25	-0.32	3.54E-02
	Rps5	-0.21	4.46E-02
	Serpine1	-0.70	4.01E-02
	Serpine2	-0.31	1.86E-03
	Sbsn	-0.71	5.48E-03
	Tnc	-0.43	1.71E-02
	Timp1	-0.84	1.54E-02
	Tgfbi	-0.38	4.74E-02
	Tgm2	-0.32	8.53E-03
	Vcan	-0.61	1.42E-02

Supplemental Table S1- continued

Enrichment Cluster	Gene	log2 Fold Change	P value
**Protease inhibitor (13 genes)**	Cstb	-0.37	1.81E-02
	Gpc3	-1.05	1.13E-04
	Lxn	-0.50	8.20E-03
	Ngf	-0.54	8.84E-04
	Serpina10	-1.07	1.66E-03
	Serpina1b	-1.06	8.91E-03
	Serpinb1a	-0.32	4.58E-03
	Serpinb6a	-0.31	6.35E-03
	Serpinb8	-0.40	1.22E-02
	Serpine1	-0.70	4.01E-02
	Serpine2	-0.31	1.86E-03
	Timp1	-0.84	1.54E-02
	Wfdc2	-0.39	2.67E-02

Enrichment Cluster	Gene	log2 Fold Change	P value
**Complement and coagulation cascade (12 genes)**	C2	-0.29	3.53E-02
	C3	-0.59	2.82E-02
	C3ar1	-0.32	2.56E-02
	Cfi	-1.16	4.46E-05
	F10	-0.75	3.66E-02
	F5	-1.09	3.29E-05
	F7	-0.83	4.98E-02
	Fgb	-1.30	4.05E-02
	Mbl1	-0.84	2.09E-02
	Pros1	-0.18	2.60E-02
	Serpina1b	-1.06	8.91E-03
	Serpine1	-0.70	4.01E-02

Enrichment Cluster	Gene	log2 Fold Change	P value
**Immunity (24 genes)**	C2	-0.29	3.53E-02
	C3	-0.59	2.82E-02
	Cd300a	-0.45	3.51E-02
	Cd300lb	-0.93	8.62E-03

Supplemental Table S1- continued

Enrichment Cluster	Gene	log2 Fold Change	P value
**Immunity (24 genes)-continued**	Cd300ld	-0.47	3.39E-02
	Cfi	-1.16	4.46E-05
	Clec4n	-0.67	1.61E-02
	Clec7a	-0.51	1.70E-02
	Dcstamp	-0.83	2.85E-02
	Ddx58	-0.19	3.18E-02
	Eif2ak2	-0.21	2.08E-02
	Fcgr1	-0.43	3.71E-02
	Fgb	-1.30	4.05E-02
	Fgr	-0.56	3.85E-02
	Il1f6	-0.96	4.33E-02
	Il34	-0.65	3.08E-03
	Lbp	-0.38	1.42E-02
	Lgals3	-0.40	3.45E-02
	Lst1	-0.50	9.65E-03
	Mbl1	-0.84	2.09E-02
	Nlrp10	-0.92	3.57E-03
	Olr1	-0.89	1.19E-02
	Smpdl3b	-0.68	2.09E-02
	Tlr2	-0.61	4.50E-04

Enrichment Cluster	Gene	log2 Fold Change	P value
**Cytokine (14 genes)**	Bmp1	-0.36	1.42E-02
	Clcf1	-0.60	1.47E-02
	Ccl17	-0.62	3.47E-02
	Ccl2	-0.65	5.16E-03
	Ccl9	-0.57	1.75E-02
	Cxcl1	-1.11	4.07E-03
	Cxcl14	-0.33	3.01E-04
	Cxcl16	-0.33	1.80E-02
	Gdf11	-0.25	4.10E-02
	Gdf15	-0.89	2.00E-02
	Il1f6	-0.96	4.33E-02
	Il34	-0.65	3.08E-03
	Lif	-1.02	2.08E-03
	Spp1	-0.91	6.45E-03

Supplemental Table S2. DAVID pathway analysis-enriched clusters of up-regulated genes in the kidney samples of Ade + IMC group compared to Ade group

Enrichment Cluster	Gene	log2 Fold Change	P value
**Lipid metabolism (29 genes)**	Aacs	0.71	2.82E-04
	Acacb	1.02	2.42E-02
	Acadm	0.78	1.43E-02
	Acadsb	0.31	4.87E-02
	Acsl1	0.60	2.66E-02
	Acsm5	0.79	1.93E-02
	Agps	0.28	3.14E-02
	Cers2	0.12	4.02E-02
	Cers4	0.26	2.20E-02
	Cyp51	0.66	2.58E-02
	Echs1	0.26	4.22E-02
	Elovl2	0.62	3.51E-02
	Elovl5	0.25	1.37E-03
	Fa2h	0.60	3.14E-03
	Fads6	0.52	1.30E-02
	Fam57b	0.79	4.66E-02
	Fasn	1.90	4.39E-02
	Gal3st1	0.30	7.05E-03
	Hadhb	0.42	1.96E-02
	Hmgcr	0.52	3.08E-02
	Hpgd	0.43	3.84E-02
	Hsd11b1	0.53	1.36E-02
	Lpl	0.89	2.03E-04
	Lypla1	0.35	4.87E-02
	Mgll	0.66	1.25E-02
	Pla2g12a	0.28	4.50E-02
	Pnpla2	0.64	1.75E-02
	Scd1	1.68	1.21E-02
	Sult1a1	0.47	2.43E-03

Supplemental Table S2- continued

Enrichment Cluster	Gene	log2 Fold Change	P value
**Mitochondria (75 genes)**	Abcc9	0.45	2.20E-02
	Abhd12	0.18	2.04E-02
	Acacb	1.02	2.42E-02
	Acadm	0.78	1.43E-02
	Acadsb	0.31	4.87E-02
	Acat2	0.39	5.25E-03
	Acsl1	0.60	2.66E-02
	Acsm5	0.79	1.93E-02
	Acyp2	0.42	2.38E-02
	Adcy10	1.68	3.90E-02
	Agps	0.28	3.14E-02
	Alas2	1.58	8.99E-08
	Aldoc	0.43	9.42E-04
	Atg13	0.19	1.30E-02
	Bcat1	0.73	9.10E-03
	Bcat2	0.29	2.07E-02
	Bckdhb	0.41	9.16E-03
	Bckdk	0.19	4.30E-03
	Bdh1	0.62	3.75E-02
	Cav1	0.41	1.81E-02
	Cd36	0.71	5.35E-03
	Ckb	0.29	3.20E-02
	Coq9	0.30	3.36E-02
	Cox8b	2.03	1.67E-02
	Dhtkd1	0.52	2.03E-02
	Dmpk	0.27	3.47E-02
	Echdc3	0.46	1.52E-02
	Echs1	0.26	4.22E-02
	Etfa	0.31	4.28E-02
	Fasn	1.90	4.39E-02
	Fbxl4	0.21	3.91E-02
	Ghr	0.62	3.48E-02
	Gtpbp3	0.22	4.30E-02
	Hadhb	0.42	1.96E-02
	Hibch	0.44	3.92E-03
	Hk1	0.22	4.11E-02
	Hsd3b2	0.96	4.57E-03
	Hsd3b3	0.44	2.89E-02
	Hspa1b	0.28	4.55E-02

S**upplemental Table S2-** continued

Enrichment Cluster	Gene	log2 Fold Change	P value
**Mitochondria (75 genes)-continued**	Ivd	0.31	4.55E-02
	Kyat3	0.83	1.15E-02
	Lpin1	0.40	1.38E-02
	Lypla1	0.35	4.87E-02
	Macrod1	0.30	4.79E-02
	Mapk10	0.81	3.42E-02
	Mccc2	0.44	4.89E-02
	Mcur1	0.29	3.08E-02
	Mmab	0.27	1.28E-02
	Mpv17l	1.24	9.71E-03
	Mrps6	0.32	9.13E-03
	Mtfr1l	0.17	4.23E-02
	Mtrf1	0.29	4.86E-02
	Mtus1	0.28	1.42E-02
	Nudt19	0.77	5.80E-04
	Pdk1	0.22	3.28E-02
	Pink1	0.28	4.37E-02
	Pmpca	0.14	3.87E-02
	Ppm1k	0.43	2.25E-02
	Ptcd2	0.22	4.44E-02
	Pxmp2	0.50	2.69E-02
	Rnf185	0.19	1.42E-02
	Sfxn5	0.30	3.40E-02
	Slc25a27	0.26	4.41E-02
	Slc25a35	0.30	3.09E-02
	Snca	1.07	2.83E-02
	Stard7	0.17	3.30E-02
	Sucla2	0.35	3.73E-02
	Tbrg4	0.19	2.89E-02
	Tfrc	0.41	1.59E-02
	Them7	0.58	2.46E-02
	Tmtc1	0.55	1.57E-02
	Tst	0.39	4.66E-02
	Ubiad1	0.41	2.62E-02
	Ucp3	2.08	3.34E-02
	Uqcrc2	0.27	3.50E-02

Supplemental Table S2- continued

Enrichment Cluster	Gene	log2 Fold Change	P value
**Ion transport (35 genes)**	Aqp2	0.76	6.18E-05
	Aqp4	1.24	2.19E-03
	Aqp6	0.61	9.32E-06
	Aspn	1.03	1.37E-03
	Atp1b2	0.20	4.94E-02
	Cacna1g	0.32	4.16E-03
	Clcnka	0.60	1.19E-03
	Cldn15	0.50	1.60E-03
	Cnnm1	0.37	1.38E-02
	Kcna1	1.16	2.08E-04
	Kcnc3	0.62	1.31E-02
	Kcng1	1.44	2.06E-02
	Kcnh6	2.55	3.25E-02
	Kcnj13	1.92	3.18E-02
	Kcnk2	0.46	1.72E-02
	Kcnk3	1.42	4.87E-02
	Kcnmb2	1.99	3.37E-02
	Kcnq4	0.64	4.18E-02
	Kcnt1	1.26	8.63E-07
	Kctd12b	0.29	2.21E-02
	Mcoln3	0.30	4.24E-02
	Mcur1	0.29	3.08E-02
	Sfxn5	0.30	3.40E-02
	Slc12a1	0.51	2.30E-02
	Slc26a7	0.68	2.71E-02
	Slc39a8	0.63	1.85E-02
	Slc4a1	0.69	7.40E-03
	Slc4a5	1.28	1.62E-05
	Slc5a6	0.66	2.54E-03
	Slc6a15	1.42	6.40E-05
	Slc9a8	0.70	2.37E-02
	Tmco3	0.37	2.09E-02
	Tmem109	0.23	8.02E-03
	Tmem38b	0.31	1.57E-02
	Trpm1	1.21	3.27E-03

Supplemental Table S2- continued

Enrichment Cluster	Gene	log2 Fold Change	P value
**Endoplastic reticulum (59 genes)**	Acsl1	0.60	2.66E-02
	Amfr	0.26	8.01E-03
	Ano5	0.65	1.10E-02
	Aoc3	0.70	1.16E-02
	Apmap	0.37	4.42E-02
	Apod	1.12	1.16E-02
	Cav1	0.41	1.81E-02
	Cd36	0.71	5.35E-03
	Cdnf	1.06	6.28E-03
	Cers2	0.12	4.02E-02
	Cers4	0.26	2.20E-02
	Ces1f	0.47	2.84E-02
	Clcnka	0.60	1.19E-03
	Col4a3	0.37	4.91E-02
	Colgalt2	0.47	2.13E-02
	Cyp51	0.66	2.58E-02
	Dio1	0.73	3.09E-02
	Dmpk	0.27	3.47E-02
	Elovl2	0.62	3.51E-02
	Elovl5	0.25	1.37E-03
	Fa2h	0.60	3.14E-03
	Fam57b	0.79	4.66E-02
	Fibin	0.69	2.03E-02
	Hadhb	0.42	1.96E-02
	Herpud1	0.34	4.00E-02
	Hmgcr	0.52	3.08E-02
	Hpd	1.47	1.97E-02
	Hsd11b1	0.53	1.36E-02
	Hsd3b2	0.96	4.57E-03
	Hsd3b3	0.44	2.89E-02
	Itga8	0.45	3.23E-02
	Kcna1	1.16	2.08E-04
	Kcnk2	0.46	1.72E-02
	Lpin1	0.40	1.38E-02
	Mfsd2a	1.30	2.38E-03
	Myo5a	0.46	1.11E-02
	Nat8	0.87	6.52E-04
	Nat8f6	0.91	4.43E-02
	Notch4	0.33	2.49E-02

Supplemental Table S2- continued

Enrichment Cluster	Gene	log2 Fold Change	P value
**Endoplastic reticulum (59 genes)-continued**	Nr3c2	0.29	4.36E-02
	Ormdl3	0.35	1.09E-02
	Pla2g12a	0.28	4.50E-02
	Ptpn5	1.19	5.34E-03
	Ric3	0.66	3.15E-03
	Rnf185	0.19	1.42E-02
	Sar1b	0.28	2.15E-02
	Scd1	1.68	1.21E-02
	Shisa2	0.72	1.05E-02
	Sil1	0.21	3.62E-02
	Tenm2	0.49	4.07E-02
	Tgfbr3	0.39	1.36E-02
	Thbs4	0.92	4.73E-03
	Tmem100	1.62	2.07E-04
	Tmem109	0.23	8.02E-03
	Tmem119	0.38	2.54E-02
	Tmem189	0.38	1.68E-02
	Tmem38b	0.31	1.57E-02
	Tmem50b	0.17	3.10E-02
	Ubiad1	0.41	2.62E-02

Enrichment Cluster	Gene	log2 Fold Change	P value
**Cellular water homeostasis (6 genes)**	Aqp1	0.40	3.44E-02
	Aqp2	0.76	6.18E-05
	Aqp3	0.49	1.03E-02
	Aqp4	1.24	2.19E-03
	Aqp6	0.61	9.32E-06
	Slc6a12	0.54	3.26E-02

Supplemental Table S3. qPCR primers used in this study

Primer	Sequence	Primer	Sequence
c-ATF4-F	CTTGATGTCCCCCTTCGACC	m-Ccl2-F	CACTCACCTGCTGCTACTCA
c-ATF4-R	GGCATCCTCCTTTCCGTTGT	m-Ccl2-R	GCTTGGTGACAAAAACTACAGC
c-CCL20-F	CATGGGCTTCACACAACAGC	m-Ccl20-F	CCAGGCAGAAGCAAGCAACTA
c-CCL20-R	CTATGGCTGAGGAGACGCAC	m-Ccl20-R	TTTGGATCAGCGCACACAGA
c-CCL2-F	TCCCAAAGAAGCTGTGATCTTCA	m-Col1a1-F	GAGAGGTGAACAAGGTCCCG
c-CCL2-R	TTTGCAGTTTGGGTTTGGCT	m-Col1a1-R	AAACCTCTCTCGCCTCTTGC
c-CHOP/DDIT3-F	CAACGAGGCTCTAAGGTCGC	m-Col3a1-F	GAGGAATGGGTGGCTATCCG
c-CHOP/DDIT3-R	TACAGTTGGATCAGGCTCTGC	m-Col3a1-R	TCGTCCAGGTCTTCCTGACT
c-GAPDH-F	TTCCACGGCACAGTCAAG	m-Cox2/Ptgs2-F	TTCAACACACTCTATCACTGGC
c-GAPDH-R	ACTCAGCACCAGCATCAC	m-Cox2/Ptgs2-R	AGAAGCGTTTGCGGTACTCAT
c-IL1A-F	TGAAGCCCAGATCAGTAGCA	m-Eif2a-F	CAACGTGGCAGCCTTACA
c-IL1A-R	CTCCCATGTCAAACTTCACTGC	m-Eif2a-R	TTTCATGTCATAAAGTTGTAGGTTAGG
c-IL8-F	ACAGTGGCCCACATTGTGAA	m-Il6-F	CAACGATGATGCACTTGCAGA
c-IL8-R	GGATCTTGTTTCTCAGCCTTCTT	m-Il6-R	TGGAAATTGGGGTAGGAAGGAC
h-B2M-F	AGATGAGTATGCCTGCCGTG	m-Lcn2-F	GGAACGTTTCACCCGCTTTG
h-B2M-R	TCATCCAATCCAAATGCGGC	m-Lcn2-R	CATTGGTCGGTGGGGACAGA
h-CCL2-F	TGCTCATAGCAGCCACCTTCA	m-Rpl13a-F	CCCTCCACCCTATGACAAGA
h-CCL2-R	GACACTTGCTGCTGGTGATTC	m-Rpl13a-R	TTCTCCTCCAGAGTGGCTGT
h-IL6-F	TTCAATGAGGAGACTTGCCTG	m-Vcam1-F	TGGAGGTCTACTCATTCCCTGA
h-IL6-R	CTGGCATTTGTGGTTGGGTC	m-Vcam1-R	GACAGGTCTCCCATGCACAA
h-IL8-F	TCTCTTGGCAGCCTTCCTGATTTC		
h-IL8-R	GTGTGGTCCACTCTCAATCACTCT		
h-TNF-a-F	GCTGCACTTTGGAGTGATCG		
h-TNF-a-R	CTTGTCACTCGGGGTTCGAG		

Primers from various species are designated as: c: canine, h: human, m: mouse

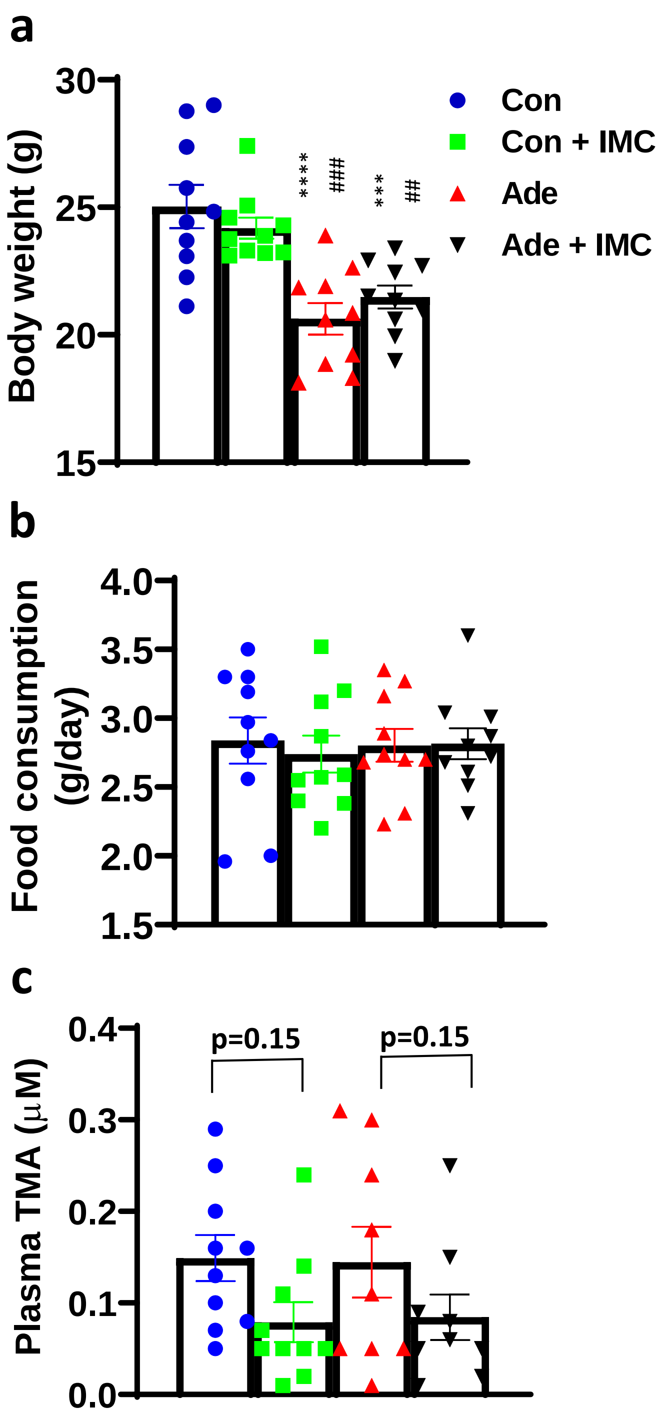

Supplemental Figure S1. Body weight (a), food consumption (b), and plasma TMA (c) data of mice included in this study. Data were obtained from the same mice described in Figure 1. Sample sizes were n = 10/group for (a) and (b), and n = 9-10/group for (c). Post hoc analysis was performed after significant two-way ANOVA. For (a) Symbols: ***: $P < 0.001$, ****: $P < 0.0001$ compared to control (Con) group. ##: $P < 0.01$, ###: $P < 0.001$ compared to control (Con) + IMC group.

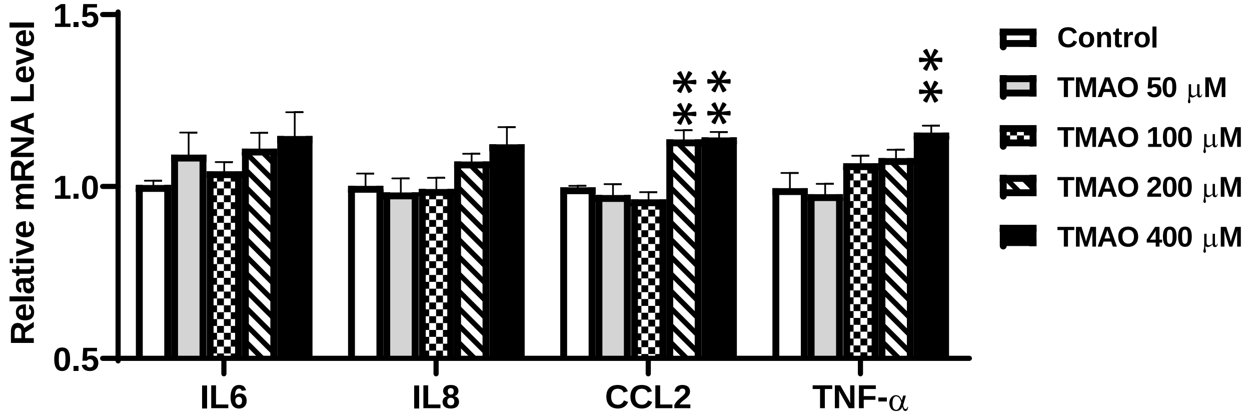

Supplemental Figure S2. TMAO treatment induces the expression of inflammatory genes in normal human primary renal proximal tubule epithelial cells, RPTEC. RPTEC cells were treated with control media or media containing various doses of TMAO (n = 4/group) for 48 hours Before examination of gene expression by qPCR. Post hoc Tukey's multiple comparisons test was performed after significant one-way ANOVA. Symbol: **: $p < 0.01$ as compared to the control group.

Supplemental Information: Full length, uncropped Images of WB in the Manuscript

Title: Inhibition of microbiota-dependent TMAO production attenuates chronic kidney disease in mice

Authors

Wenchao Zhang, Aika Miikeda, Jonathan Zuckerman, Xun Jia, Sarada Charugundla, Zhiqiang Zhou, Karolina Elżbieta Kaczor-Urbanowicz, Clara Magyar, Fangfei Guo, Zeneng Wang, Matteo Pellegrini, Stanley L. Hazen, Susanne B. Nicholas, Aldons J. Lusis, and Diana M. Shih

Description

In the supplemental information, the original images of Figure 2e are shown without “auto-contrast adjustment” of Adobe Photoshop software.

Figure 2e (uncropped images)

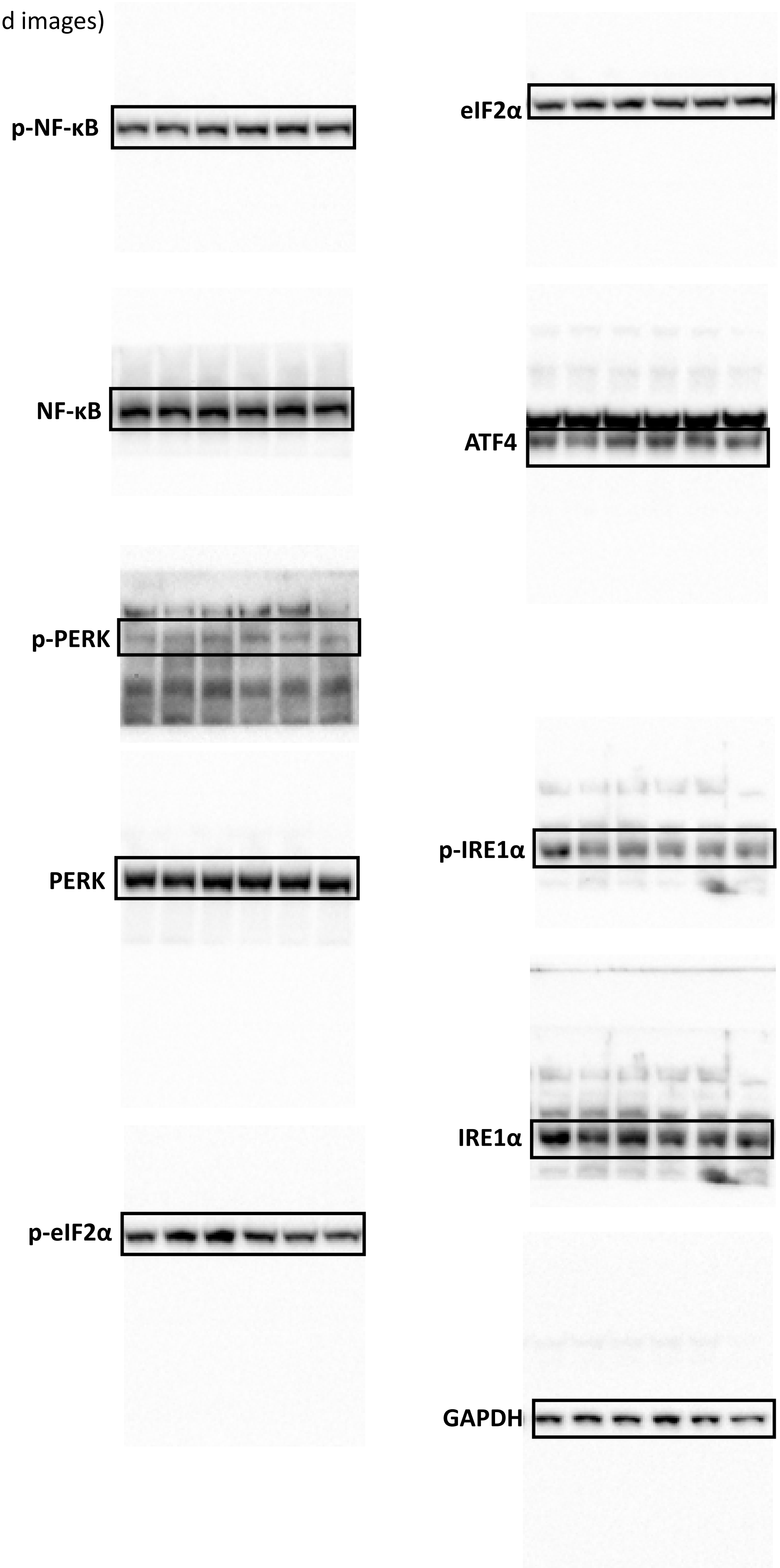

Supplement: Supplementary file 1 — Supplementary Information [file 41598_2020_80063_MOESM1_ESM.pdf]
